# The Transcription Factor StuA Regulates the Glyoxylate Cycle in the Dermatophyte *Trichophyton rubrum* under Carbon Starvation

**DOI:** 10.3390/ijms25010405

**Published:** 2023-12-28

**Authors:** Monise Fazolin Petrucelli, Leonardo Martins-Santana, Pablo R. Sanches, Vanderci M. Oliveira, Antonio Rossi, Nilce M. Martinez-Rossi

**Affiliations:** Department of Genetics, Ribeirão Preto Medical School, University of São Paulo, Ribeirão Preto 14049-900, SP, Brazil; mofazolin@gmail.com (M.F.P.); leonardo.lms95@gmail.com (L.M.-S.); psanches@usp.br (P.R.S.); cuca@fmrp.usp.br (V.M.O.); anrossi@usp.br (A.R.)

**Keywords:** dermatophytes, *Trichophyton rubrum*, metabolism, glyoxylate cycle, APSES transcription factors, StuA, intron retention, carbon source, alternative splicing

## Abstract

*Trichophyton rubrum* is the primary causative agent of dermatophytosis worldwide. This fungus colonizes keratinized tissues and uses keratin as a nutritional source during infection. In *T. rubrum*–host interactions, sensing a hostile environment triggers the adaptation of its metabolic machinery to ensure its survival. The glyoxylate cycle has emerged as an alternative metabolic pathway when glucose availability is limited; this enables the conversion of simple carbon compounds into glucose via gluconeogenesis. In this study, we investigated the impact of *stuA* deletion on the response of glyoxylate cycle enzymes during fungal growth under varying culture conditions in conjunction with post-transcriptional regulation through alternative splicing of the genes encoding these enzymes. We revealed that the Δ*stuA* mutant downregulated the malate synthase and isocitrate lyase genes in a keratin-containing medium or when co-cultured with human keratinocytes. Alternative splicing of an isocitrate lyase gene yielded a new isoform. Enzymatic activity assays showed specific instances where isocitrate lyase and malate synthase activities were affected in the mutant strain compared to the wild type strain. Taken together, our results indicate a relevant balance in transcriptional regulation that has distinct effects on the enzymatic activities of malate synthase and isocitrate lyase.

## 1. Introduction

Efficient nutrient assimilation by pathogenic fungi during infection is crucial for survival [1,2]. The dermatophyte *Trichophyton rubrum* infects and degrades keratinized tissues, such as nails and skin, by breaking down proteins into free amino acids and peptides [3,4,5]. Fungi assimilate these products as carbon sources via membrane transporters [6,7]. Upon infection, the fungus adapts to the host milieu using molecular mechanisms that facilitate its metabolic flexibility, colonization, and invasion [8]. Orchestrated mechanisms of gene modulation and interactions with transcription factors govern the balance of metabolic reprogramming. This balance contributes to fungal fitness and pathogenicity and represents an attractive target for prospecting antifungal drugs [6,9].

During metabolic adaptation in fungal pathogenesis, the glyoxylate cycle is pivotal for the use of alternative carbon sources. Under glucose deprivation, fungal pathogens undergo a metabolic transition toward the glyoxylate cycle. This adaptation enables them to assimilate two carbon compounds [10]. Fungal cells mobilize fatty acids to generate acetyl-CoA, which activates the glyoxylate cycle. In this metabolic cycle, isocitrate lyase converts isocitrate into glyoxylate, and malate synthase converts glyoxylate into malate. Both enzymes are exclusive to this cycle [11,12]. Malate is then driven toward oxaloacetate production and continually reacts with acetyl-CoA to maintain the cycle. Succinate is shuttled to the tricarboxylic acid (TCA) cycle in mitochondria, where it is metabolized into cycle intermediates to generate oxaloacetate. Oxaloacetate molecules transported to the cytosol trigger gluconeogenesis and reestablish the glucose supply in fungal cells [1].

Transcription factors (TFs) play a pivotal role in signaling pathways by orchestrating mechanisms that either activate or suppress molecular responses based on the specific biological context to which the fungus is exposed [13,14,15]. The APSES family belonging to transcription factors of the basic helix–loop–helix (bHLH) class, which includes Asm1p, Phd1p, Sok2p, Efg1p, and StuA, is unique to fungi and plays an essential role in regulating a wide range of processes, including fungal growth, virulence, pathogenicity, and metabolism [16,17,18]. In *Aspergillus fumigatus*, StuA is critical for the morphogenesis and biosynthesis of secondary metabolites [19,20]. Additionally, studies involving null mutants of *stuA* in the dermatophyte *Arthroderma benhamiae* have demonstrated its involvement in keratin degradation and sexual development [21]. Our recent studies demonstrated the role of StuA in several aspects involved in the virulence of *T. rubrum* [22]. In a previous RNA sequencing analysis [23] of transcripts generated by the Δ*stuA* strain during growth on glucose or keratin, the impact of StuA deletion on central carbon metabolism was evident reducing transcript levels of the genes encoding the glyoxylate cycle enzymes [23]. This result raised hypotheses about whether StuA would also regulate the enzymatic activity of glyoxylate cycle enzymes during fungal–host interaction [24,25,26].

Post-transcriptional regulation through alternative splicing (AS) allows the production of various protein isoforms in response to physiological requirements and environmental cues, often serving as drivers of phenotypic diversity within the eukaryotic cell proteome [27,28,29]. Intron retention (IR) is one of the most common AS events in fungi [30,31], and it may be relevant in the regulatory mechanisms of fungal physiology, adaptation to fungal niches, pathogenicity, and drug resistance [13,32,33].

In this study, we hypothesized that the transcription factor StuA plays a significant role in regulating essential enzymes of the glyoxylate cycle depending on the carbon source or in an infection-like scenario. We also investigated the possibility of post-transcriptional regulation through IR events in the transcripts of malate synthase and isocitrate lyase genes. Post-transcriptional regulation is a crucial mechanism that facilitates fungal adaptation, particularly under glucose-depleted conditions. Our findings suggested that StuA regulates the transcription of the main enzymes of the glyoxylate cycle. We also showed that modulation of the isoforms generated by the AS of an isocitrate lyase gene depended on culture conditions.

## 2. Results

### 2.1. Reannotation of Isocitrate Lyase as a Single Gene (OR643895)

By sequencing the DNA and cDNA of the exonic and their flanking regions of the TERG_11637, TERG_11638, and TERG_11639, we concluded that they comprised a single gene identified as OR643895 (Figures S1 and S2). Multiple alignments of the OR643895 nucleotide sequence with the isocitrate lyase-coding genes and protein sequences from various dermatophytes showed a homology of more than 95% with the isocitrate lyase-coding genes of *Trichophyton tonsurans*, *Trichophyton verrucosum*, *Trichophyton equinum*, *Arthroderma benhamiae*, and *Microsporum canis*. Therefore, *T. rubrum* has only two genes encoding isocitrate lyase: TERG_01271 and OR643895.

### 2.2. The ΔstuA Mutant Reduces the Transcription of Isocitrate Lyase and Malate Synthase Genes

The wild type (WT) strain exhibited an approximately 10-fold upregulation of genes encoding isocitrate lyase during fungal growth in keratin compared to growth in glucose. The protein StuA exerted distinct regulatory effects on the modulation of isocitrate lyase during the cultivation of the Δ*stuA* strain in glucose. While OR643895 transcripts were downregulated at 24 and 48 h, TERG_01271 transcripts were derepressed at 24 h and stayed at the same level in transcript abundance at 48 h compared to the WT (control) strain. Transcript levels of TERG_01281 also exhibited a decline in the Δ*stuA* strain at both time points.

**Figure 1:**
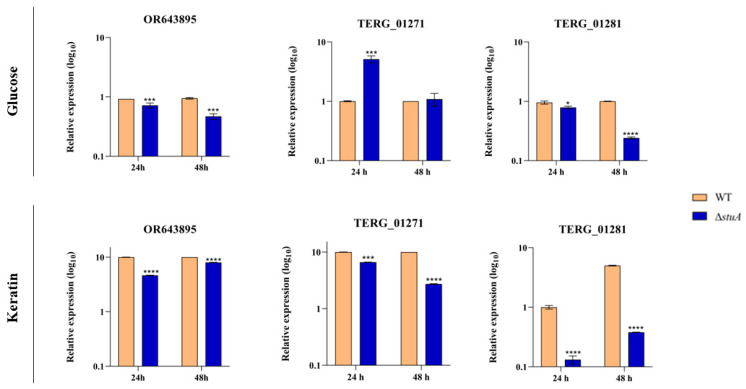
Relative expression analysis of isocitrate lyase (OR643895 and TERG_01271) and malate synthase (TERG_01281) transcripts in wild type (WT) and Δ*stuA* mutant strains during growth on glucose or keratin. The WT strain served as the control. Statistical significance was determined using an unpaired Student’s *t*-test with Holm–Sidak correction for multiple comparisons. * *p* < 0.05, *** *p* < 0.001, and **** *p* < 0.0001.

Fungal growth in keratin resulted in reductions in the transcript levels of isocitrate lyase and malate synthase gene isoforms in the Δ*stuA* strain across all evaluated time points (Figure 1).

**Figure 2:**
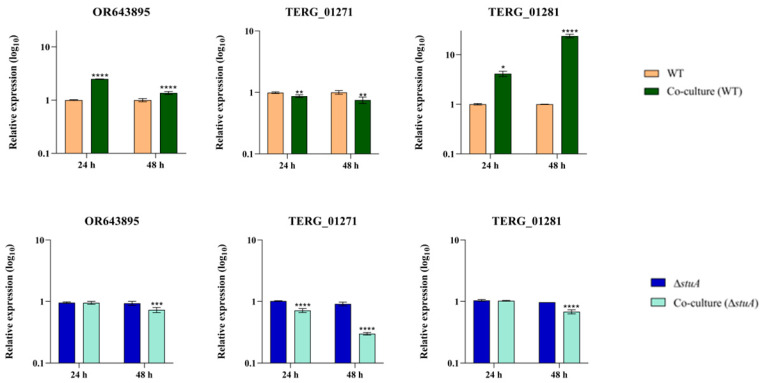
Relative expression analysis of isocitrate lyase (OR643895 and TERG_01271) and malate synthase (TERG_01281) transcripts in wild type (WT) and Δ*stuA* mutant strains during co-culture with HaCaT keratinocytes. The WT and Δ*stuA* strains without keratinocytes were used as the controls in their respective co-culture assays. Statistical significance was determined using an unpaired Student’s *t*-test with Holm–Sidak correction for multiple comparisons. * *p* < 0.05, ** *p* < 0.01, *** *p* < 0.001, and **** *p* < 0.0001.

We conducted transcriptional analysis of genes encoding isocitrate lyase and malate synthase in an infection-like scenario in human keratinocytes. Our results revealed different levels of isocitrate lyase (OR643895 and TERG_01271) transcripts during co-culture with the WT strain. We observed an overexpression of OR643895 at 24 and 48 h, whereas TERG_01271 exhibited lower transcript levels during the same period. The gene encoding malate synthase (TERG_01281) was upregulated at 24 and 48 h. During co-culture with the Δ*stuA* strain, we observed a reduced expression of OR643895 and TERG_01281 only at 48 h post-infection. However, TERG_01271 exhibited decreased transcript levels at 24 and 48 h (Figure 2).

### 2.3. Alternative Splicing Assay

**Figure 3:**
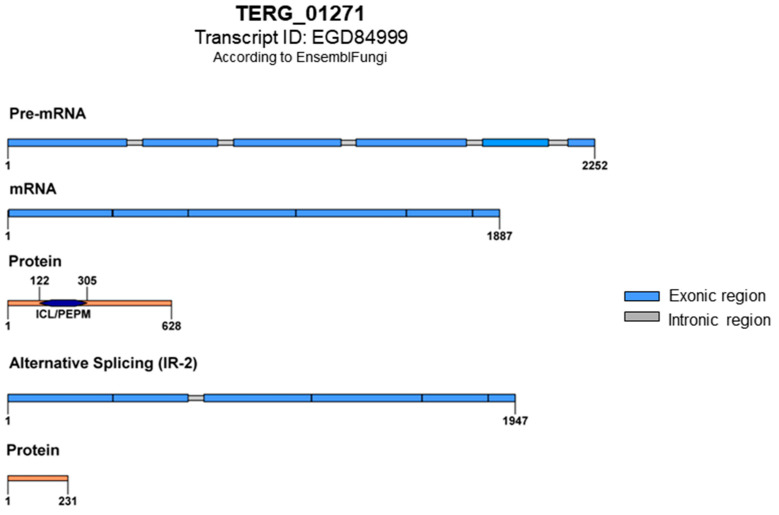
Schematic representation of TERG_01271 with conventional splicing and protein translation featuring the ICL/PEPM domains (Isocitrate Lyase-like/Penta-EF-hand Protein Motif). The ICL domain is responsible for the catalytic activity of isocitrate lyase, and the PEPM domain is associated with calcium-binding activity. An alternative splicing event with intron 2 retention results in an mRNA with premature stop codons and the formation of a putative truncated protein; both domains lost.

In a previously generated RNA sequencing dataset of the Δ*stuA* mutant grown in glucose and keratin published by our research group [23], we observed AS events in TERG_01271. Our in silico analysis of the protein sequence and conserved motifs in this gene revealed that AS generated mRNAs with premature stop codons that disrupted protein translation. The retention of intron 2 (IR-2) resulted in a putative protein with 231 amino acid residues lacking conserved domains (Figure 3).

**Figure 4:**
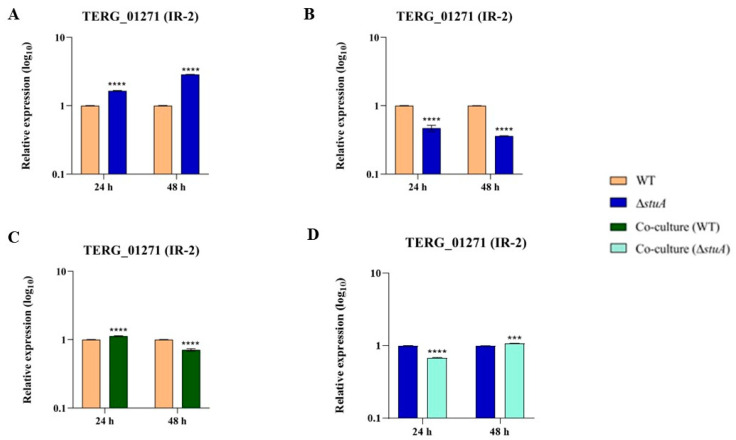
Relative expression analysis of TERG_01271 transcripts with IR-2 in the WT (control) and Δ*stuA* strains. Expression patterns are shown during growth in glucose (**A**) and keratin (**B**) media, as well as during co-culture with human keratinocytes for the WT (**C**) and Δ*stuA* (**D**) strains. The WT and Δ*stuA* strains without keratinocytes were used as controls in their respective co-culture assays. Statistical significance was determined using an unpaired Student’s *t*-test with Holm–Sidak correction for multiple comparisons. *** *p* < 0.001, **** *p* < 0.0001.

We identified IR-2 events in TERG_01271 during the growth of the WT and Δ*stuA* strains in glucose and keratin, as well as during co-culture with human keratinocytes. We noted increased expression of the TERG_01271 isoform transcripts with IR-2 in the mutant strain compared to the WT strain when grown in glucose (Figure 4A). However, IR-2 transcript levels decreased in the mutant when both strains were cultured in a keratin medium (Figure 4B). During co-culture, IR-2 transcript levels exhibited a distinct profile depending on the duration of the interaction between the fungus and the host. In the WT strain, we observed an increased number of IR-2 transcripts at 24 h, followed by a significant decrease in the expression of this alternative isoform at 48 h when co-cultured with human keratinocytes (Figure 4C). However, for the mutant Δ*stuA*, we noted that the IR-2 transcripts were initially repressed at 24 h but induced at 48 h of co-culture (Figure 4D).

### 2.4. The Enzymatic Activities of Isocitrate Lyase and Malate Synthase during Fungal Growth in a Medium Supplemented with Glucose or Keratin

**Figure 5:**
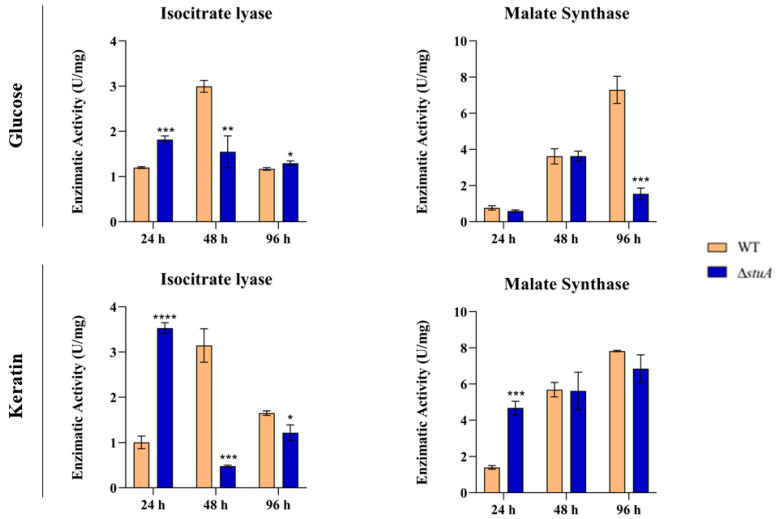
Enzymatic activities of isocitrate lyase and malate synthase in the WT and Δ*stuA* mutant strains during growth in media containing glucose or keratin. Statistical significance was determined using an unpaired Student’s *t*-test with Holm–Sidak correction for multiple comparisons. * *p* < 0.05, ** *p* < 0.01, *** *p* < 0.001, and **** *p* < 0.0001.

The enzymatic activity of isocitrate lyase was notably higher in glucose and keratin in the Δ*stuA* mutant after 24 h compared to the WT control. However, after 48 h, we noted a significant increase in enzymatic activity in the WT strain under both growth conditions, whereas the mutant strain grown on keratin exhibited a substantial reduction in activity. At 96 h, we observed a distinct pattern of enzymatic activity during the growth of the mutant depending on the nutritional carbon source (Figure 5).

Regarding the enzymatic activity of malate synthase, we observed a substantial decrease in the Δ*stuA* strain compared to the control after 96 h of growth in glucose. However, during keratin growth, we observed higher enzymatic activity in the mutant at 24 h, with no statistically significant differences in enzymatic activity observed between the mutant and WT during the remaining time intervals (Figure 5).

Table 1 summarizes our main findings in an attempt to correlate the read count numbers obtained in our previously published RNA sequencing dataset [23] with the expression analysis of genes involved in the glyoxylate cycle and their respective enzyme activities in the WT and Δ*stuA* strains.

## 3. Discussion

Under glucose-deprived conditions, fungal metabolism relies on carbon acquisition from alternative nutritional sources. One potential approach involves the activation of the glyoxylate cycle. Given its absence in humans, this pathway is an attractive and promising target for antifungal development, primarily because of its exclusive non-human enzymes, isocitrate lyase, and malate synthase [1]. These enzymes become active in the dermatophyte *T. rubrum* when grown on keratin or when exposed to cytotoxic drugs in an infection-like scenario involving human keratinocytes [6,34,35,36,37,38]. For the first time, we present evidence of the transcriptional modulation of essential enzyme-coding genes within the glyoxylate cycle by StuA. Through DNA sequencing, we concluded that the three exonic regions of the genome (TERG_11637, TERG_11638, and TERG_11639), previously annotated as separate entities, were part of the same gene, and have now been identified as OR643895. Furthermore, our in silico analysis showed the presence of a StuA consensus-binding site in the TERG_01271 promoter region, implying possible direct transcriptional regulation of this gene by StuA in *T. rubrum.* Also, the StuA consensus-binding site is conserved in the promoter region of the TERG_01271 homolog in other dermatophytes (Figure S3).

### 3.1. The Expression of Glyoxylate Cycle Genes Depends on StuA during Fungal Growth in Keratin

The regulation of the expression of glyoxylate cycle genes relies on the transcriptional control exerted by transcription factors. Here, we show that when considering fungal growth in keratin, the absence of StuA significantly reduces the transcription levels of both isocitrate lyase and malate synthase genes (Figure 1). Notwithstanding, our results presented a distinct transcriptional regulation pattern in glucose cultures for isocitrate lyase-coding genes, suggesting that glucose might alter the StuA-mediated regulation of glyoxylate-coding genes. However, we detected a tendency of the upregulation of TERG_01271 in the Δ*stuA* strain (for 24 and 48 h), which suggests that StuA, in wild type conditions, might have a role in repressing a relevant enzyme of the glyoxylate cycle (Figure 1). In this sense, StuA deletion promotes a cascade of stress response events that affect central carbon metabolism. A high-throughput transcriptomic analysis suggested that the Δ*stuA* strain tended to upregulate specific glutamate metabolism genes in keratin cultures [23]. Considering the repression of essential genes for the glyoxylate cycle in the Δ*stuA* strain, we infer that StuA is necessary for *T. rubrum* survival adaptation in glucose-depleted conditions.

### 3.2. Isocitrate Lyase and Malate Synthase Genes Are Upregulated during Co-Culture with Human Keratinocytes, but the Absence of StuA Impairs Their Expression

We observed the overexpression of OR643895, whereas the opposite occurred with TERG_01271 (Figure 2) in the WT strain co-culture, suggesting a dual transcriptional regulation pattern for isocitrate lyase-coding genes. However, in the Δ*stuA* strain co-cultured with human keratinocytes, the expression of the isocitrate lyase and malate synthase-coding genes was repressed, mainly after 48 h of interaction. The differences observed in the transcriptional modulation of genes encoding isocitrate lyase led us to propose that another level of transcriptional regulation drives the expression of TERG_01271 or OR643895.

Fungi trigger the glyoxylate cycle upon contact with macrophages [39,40,41,42]. This is a remarkable pathogenic strategy for determining how metabolic flexibility contributes to the virulence of fungal pathogens. In *T. rubrum*, the overexpression of genes encoding malate synthase and isocitrate lyase has been observed during a dual RNA sequencing analysis of *T. rubrum* co-cultured with human keratinocytes [36]. Here, we propose that StuA plays a significant role in activating the glyoxylate cycle in human keratinocytes, suggesting that this regulatory protein might be a potential target for impairing the virulence of *T. rubrum*.

### 3.3. Post-Transcriptional Regulation of TERG_01271 by Alternative Splicing

We observed through in silico analysis that IR events in TERG_01271 resulted in the introduction of a premature stop codon, forming a potentially putative non-functional truncated protein (Figure 3). In addition, the carbon source may influence the transcript levels of the IR isoform in *T. rubrum* via a mechanism mediated by StuA (Figure 4A,B). Our results showed that StuA represses both the TERG_01271 conventional and IR isoforms in glucose cultures but acts as an activator of identical isoforms in keratin cultures. This is reasonable considering that glyoxylate cycle genes are activated in glucose-deprived environments. Remarkably, both the carbon source and StuA may influence the AS of the glyoxylate gene in *T. rubrum*. Furthermore, the presence of mature conventional transcripts did not impair the presence of the IR isoforms in *T. rubrum*, as reported previously [13].

The balance between mutual and non-exclusive splicing isoform abundance was also observed in co-culture assays, where a challenge with keratinocytes elicited a distinct pattern for the TERG_01271 IR isoform. Although we detected IR isoforms in both WT and Δ*stuA* strain cultures (control), contact with keratinocytes triggered differences in the abundance of AS isoforms (Figure 4C,D), in contrast to the single repression pattern generated in co-cultures of conventional splicing isoforms. As the co-culture mimics an infection-like scenario, we hypothesized that the differences between conventional splicing and AS isoforms agree with the biological requirements imposed on *T. rubrum* to fight host defense strategies.

### 3.4. Isocitrate Lyase and Malate Synthase Activities Are Independently Regulated during T. rubrum Culture in Glucose or Keratin

The absence of StuA compromised isocitrate lyase activity in specific instances. We observed a higher enzymatic activity in the absence of StuA after the first 24 h of culture, regardless of the carbon source. Conversely, the mutant strain presented lower enzymatic activity after 48 h and showed distinct enzymatic activity at 96 h, depending on the carbon source. We hypothesized that throughout fungal growth (48 and 96 h), the absence of this transcription factor, which is associated with AS events, would significantly reduce the enzymatic activity of isocitrate lyase. We also observed a statistically significant increase in malate synthase enzymatic activity after 24 h of Δ*stuA* culture in keratin, followed by a reduction in activity after 96 h in glucose (Figure 5). Malate synthase is responsible for converting acetyl-CoA and glyoxylate into malate. Malate synthase activity was not affected by the reduced isocitrate lyase activity in certain instances. Therefore, even with a reduction in isocitrate lyase activity, which consequently reduces glyoxylate production, the glyoxylate generated under the conditions evaluated in this study was sufficient to stimulate malate synthase activity.

From the data summarized in Table 1, we observed compensatory activity in the expression of TERG_01271 and OR643895 in the Δ*stuA* strain, which may result in few changes in isocitrate lyase enzymatic activity. Fluctuations in read counts in the mutant strain at 24, 48, and 96 h did not correlate with significant changes in isocitrate lyase activity. We observed a similar trend in TERG_01281 expression. During growth on keratin, Δ*stuA* exhibits lower read counts compared to WT. However, this strain has shown significant isocitrate lyase activity in some instances. Additionally, the reduction in isocitrate lyase gene read counts in Δ*stuA* from 48 to 96 h did not significantly affect enzymatic activity.

It is well known that post-translational modifications can influence the catalytic potential of enzymes, including phosphorylation [43]. In *Saccharomyces cerevisiae* [44] and *Paracoccidioides brasiliensis* [45], phosphorylation has been shown to reduce isocitrate lyase activity, leading to enzyme inactivation. By analogy, we hypothesize that a similar phenomenon occurs in *T. rubrum*. However, further research is necessary to fully understand the post-translational mechanisms that govern isocitrate lyase activity in *T. rubrum*.

## 4. Materials and Methods

### 4.1. Fungal Strains and Culture Conditions

The *T. rubrum* strain CBS118892 (Westerdijk Fungal Biodiversity Institute, Utrecht, The Netherlands) was used as a reference (WT). We also used the previously constructed null mutant strain, Δ*stuA* [22]. The strains were grown on a solid malt extract agar medium (2% glucose, 2% malt extract, 0.1% peptone, 2% agar, pH 5.7) at 28 °C for 20 days. Next, to prepare a conidial suspension, we flooded the plates with a 0.9% sterile NaCl solution and filtered them through fiberglass to remove hyphal fragments. Conidial concentration was estimated using a Neubauer chamber. Then, we inoculated approximately 1 × 10^6^ conidia of each strain into 100 mL of Sabouraud dextrose broth and incubated the cultures at 28 °C for 96 h in an orbital shaker with agitation (120 rpm).

The resulting mycelia were transferred into 100 mL of a minimal medium [46] containing 70 mM sodium nitrate (Sigma-Aldrich, St. Louis, MO, USA), 50 mM glucose (Sigma-Aldrich, St. Louis, MO, USA), or 0.5% bovine keratin (*m*/*v*). We incubated the cultures for 24, 48, and 96 h at 28 °C with constant agitation (120 rpm). Subsequently, we filtered the biological material from three independent replicates of glucose or keratin cultures at each time point and stored it at −80 °C until RNA extraction.

### 4.2. Co-Culture of Fungal Strains and Human Keratinocytes

The immortalized HaCaT human keratinocyte cell line (Cell Lines Service GmbH, Eppelheim, Germany) was cultured in an RPMI-1640 medium (Sigma-Aldrich, St. Louis, MO, USA) supplemented with 10% fetal bovine serum at 37 °C in a humidified atmosphere containing 5% CO_2_. We added penicillin (100 U/mL) and streptomycin (100 µg/mL) to prevent culture medium contamination. The co-culture assays of the fungal WT or Δ*stuA* strains with HaCaT keratinocytes were performed as previously described [13]. We used uninfected keratinocytes and WT or Δ*stuA* conidia as the controls. The assay was performed in triplicate.

### 4.3. RNA Extraction and cDNA Synthesis

Total RNA was extracted using an Illustra RNAspin Mini Isolation Kit (GE Healthcare, Chicago, IL, USA), according to the manufacturer’s instructions. For fungal cell wall disruption in the co-culture, the samples were treated with a solution of lysing enzymes from *Trichoderma harzianum*, as previously described [36]. RNA concentration and purity were assessed using a NanoDrop ND-100 spectrophotometer (Thermo Fisher Scientific, Waltham, MA, USA).

Total RNA was treated with DNase I (Sigma-Aldrich, St. Louis, MO, USA) to prevent genomic DNA contamination. Subsequently, cDNA synthesis was performed using the Platus Transcriber RNase H-cDNA First Strand Kit (Sinapse Inc., Miami, FL, USA), according to the manufacturer’s instructions. To assess the quality of the obtained cDNAs, we conducted a PCR reaction using oligonucleotides to amplify a region of the constitutive β-tubulin gene, followed by analysis on an agarose electrophoresis gel. We suspended the cDNAs in 70 ng/µL dilutions for a reverse-transcription quantitative polymerase chain reaction (RT-qPCR).

### 4.4. Genomic DNA and cDNA Sequencing of Isocitrate Lyase

Previous RNA sequencing performed by our group showed aligned reads in the intergenic regions of TERG_11637, TERG_11638, and TERG_11639, all of which were annotated as encoding isocitrate lyases. The web server Augustus (https://bioinf.uni-greifswald.de/augustus/. Accessed in April 2023) was used [47] to predict genes from this supercontig region. The predicted gene sequence was aligned against the Ensembl Fungi database using BLAST tools to verify the homology among the genes of other species. The coordinates of the exons and introns were obtained by aligning the isocitrate lyase sequences from dermatophytes in the database against the predicted gene sequence.

For DNA sequencing, we designed specific primers flanking the supercontig regions of these three genes and used genomic DNA and cDNA samples (Table S1). We purified the PCR products using the Wizard^®^ SV Gel and PCR Clean-UP System Protocol (Promega Corporation, Madison, WI, USA), according to the manufacturer’s instructions. A NanoDrop ND-100 spectrophotometer (Thermo Fisher Scientific, Waltham, MA, USA) was used to assess the purity and integrity of PCR products before sequencing.

DNA sequencing was performed using a BigDye Terminator v3.1 Cycle Sequencing Kit (Applied Biosystems, Waltham, MA, USA), according to the manufacturer’s instructions, with the Sanger methodology in an ABI 3500xL Genetic Analyzer (Thermo Fisher Scientific, Waltham, MA, USA). Sequencing Analysis Software v.5.4 was used to analyze the quality of the rendered sequences. The gDNA and cDNA sequences were assembled and analyzed using DNASTAR SeqMan Ultra software (https://www.dnastar.com. Assessed in August 2023). The obtained nucleotide sequence corresponds to a single isocitrate lyase and is available in GenBank under accession number OR643895.

### 4.5. Alternative Splicing Analyses

Sequencing reads from a previous RNA sequencing analysis available at the Gene Expression Omnibus under accession numbers GSE163357 and GSE134406 [23] were mapped to the *T. rubrum* reference genome using the STAR aligner [48]. To identify AS events, we processed the aligned reads using the ASpli package in R software version 4.3.1 [49]. Differential expression was analyzed using the DESeq2 Bioconductor package [50]. The Benjamini–Hochberg-adjusted *p*-value was set to 0.05, with a Log_2_Fold Change of ±1.5 to identify the abundance of significantly modulated levels of transcripts [23].

We used in silico tools to identify the isoforms, reading frames, conserved sites, and domains of the isocitrate lyase encoded by TERG_01271 during AS events with IR and conventional splicing mRNA processing. The ExPAsy Translate Tool [51] was used to identify the translated protein sequences of the analyzed transcripts. We searched for protein domains in virtual databases such as Ensembl Fungi [52], Interpro [53], and PANTHER [54,55]. We drew a graphical representation of each isoform using Illustrator for Biological Sequences software (IBS 1.0) [56].

### 4.6. RT-qPCR Analyses

We used a QuantStudio 3 Real-Time PCR System (Applied Biosystems, Waltham, MA, USA) with the primers listed in Supplementary Table S1 for transcript quantification. For TERG_01271, which exhibited both conventional and AS events, we designed primers flanking only exon–exon junctions for traditional splicing analysis and primers within the intronic region for IR events. The concentration of each primer was standardized for reaction efficiencies between 90% and 110%. Reactions were prepared using Power SYBR™ Green PCR Master Mix (Applied Biosystems, Waltham, MA, USA) with ROX dye as a fluorescent normalizer [57]. We used the 2^−ΔΔCt^ method [58] for relative expression analysis, considering the *T. rubrum* gene *gapdh* as an endogenous control. Relative expression normalization of conventional splicing and IR events in the WT and mutant strains grown in glucose or keratin and co-cultured with human keratinocytes was performed as described previously [13]. The results are presented as the mean relative expression values from three independent replicates with standard deviations.

### 4.7. Enzymatic Activity Assays

We used the macerated mycelium of WT and Δ*stuA* strains to assess enzymatic activity. Approximately 0.75 g of macerated mycelium was mixed with 500 μL of a Tris-HCl buffer (50 mM Tris-HCl, 2 mM MgCl_2_, 2 mM dithiothreitol, pH 8.0). The samples were vortexed and centrifuged for 30 min at 1.268× *g* at 4 °C [9]. The supernatant (protein extract) was collected and stored at −80 °C until enzymatic assays for isocitrate lyase and malate synthase were performed. Proteins were quantified using the Bradford reagent (Sigma-Aldrich, St. Louis, MO, USA), and concentrations were determined using a standard curve of serial dilutions of Bovine Serum Albumin (BSA) (Sigma-Aldrich, St. Louis, MO, USA).

Isocitrate lyase activity was measured using a phenylhydrazone-based assay [41]. The reaction was initiated by adding the isocitrate substrate (potassium phosphate 50 mM, pH 7.0; MgCl_2_ 2 mM, phenylhydrazone 10 mM, D-L isocitrate 2 mM, dithiothreitol 2 mM) with 1 μg of protein extract and incubated for 15 min at 30 °C. The glyoxylate–phenylhydrazone product was quantified at 324 nm (Ɛ 16.8 mM^−1^ cm^−1^) using an Evolution™ 201 UV-Visible Spectrophotometer (Thermo Fisher Scientific, Waltham, MA, USA) [9]. Under the assessed conditions, one enzyme unit was defined as the amount of enzyme that produced 1 mmol glyoxylate–phenylhydrazone per minute.

For the enzymatic assay of malate synthase, 1 μg of protein extract was mixed with malate substrate (imidazole buffer 30 mM; pH 8.0, MgCl_2_ 10 mM, acetyl-CoA 0.25 mM, glyoxylic acid 1 mM, 5,5′-dithio-bis, 2-nitrobenzoic acid 0.2 mM) and incubated for 5 min at 30 °C. The 5-thio-2-nitrobenzoic acid + CoA derivative product was quantified at 412 nm (Ɛ 13.6 mM^−1^ cm^−1^) using an Evolution™ 201 UV-Visible Spectrophotometer (Thermo Fisher Scientific, Waltham, MA, USA) as previously described [59,60]. Under the assessed conditions, one enzyme unit produced 1 μmole of acetyl-CoA per minute in the presence of glyoxylate.

The enzymatic activities of isocitrate lyase and malate synthase were represented as units per milligram (U/mg) of total protein extract. Three biological replicates were used in each experiment.

### 4.8. Statistical Analysis

We used an unpaired *t*-test to statistically analyze the transcript quantifications and enzymatic assay results. Statistical significance was determined using the Holm–Sidak method with *p* < 0.05. Significance is represented in the graphs as * *p* < 0.05, ** *p* < 0.01, *** *p* < 0.001, and **** *p* < 0.0001. GraphPad Prism software v.6 (GraphPad Software, San Diego, CA, USA) [61] was used for statistical analysis and graph design.

## 5. Conclusions

In summary, our results provide new insights into the annotation of isocitrate lyase genes. This is the first study to report the association of the transcription factor StuA with the transcriptional regulation of genes involved in the glyoxylate cycle in conjunction with AS events. The absence of StuA impaired the expression of genes encoding isocitrate lyase and malate synthase during growth in different carbon sources and co-culture with human keratinocytes. Therefore, this transcription factor can directly regulate TERG_01271 and indirectly regulate the OR643895 and malate synthase (TERG_01281) genes. We also revealed a balance between conventional and AS in the post-transcriptional regulation of TERG_01271. Finally, we demonstrated the impairment of isocitrate lyase activity in the mutant strain under certain conditions. However, the enzymatic activity of malate synthase was not entirely affected during fungi growth in different carbon sources.

## Figures and Tables

**Table 1:** Read counts, gene expression, and enzymatic activity of genes from the glyoxylate cycle in Δ*stuA* and WT strains of *T. rubrum* grown on glucose or keratin.

**Glucose**						
**Time**	**Strain**	**Gene**	**Gene Product**	Read Counts *	**Fold Change ¥**	**Enzymatic Activity (U/mg)**
24 h	WT	TERG_01271	Isocitrate lyase	431.98 ± 26	1 ± 0.0002	1.198 ± 0.02
		OR643895		278.18 ± 36	0.952 ± 0.060	
	Δ*stuA*	TERG_01271	Isocitratetter e lyase	593.87 ± 69	5.120 ± 0.69	1.817 ± 0.08
		OR643895		147.71 ± 36	0.72 ± 0.07	
	WT	TERG_01281	Malate synthase	1229.57 ± 846	1 ± 0.060	0.765 ± 0.12
	Δ*stuA*	TERG_01281	Malate synthase	1204.53 ± 297	0.787 ± 0.040	0.598 ± 0.060
48 h	WT	TERG_01271	Isocitrate lyase	667.73 ± 302	1 ± 0.0002	2.996 ± 0.13
		OR643895		266.15 ± 57	1 ± 0.0003	
	Δ*stuA*	TERG_01271	Isocitrate lyase	496.28 ± 47	1.090 ± 0.265	1.548 ± 0.35
		OR643895		167.06 ± 36	0.47 ± 0.05	
	WT	TERG_01281	Malate synthase	5201.18 ± 2131	1 ± 0.010	3.618 ± 0.42
	Δ*stuA*	TERG_01281	Malate synthase	720.99 ± 134	0.241 ± 0.010	3.632 ± 0.27
96 h	WT	TERG_01271	Isocitrate lyase	622.49 ± 53	-	1.167 ± 0.03
		OR643895		327.91 ± 28	-	
	Δ*stuA*	TERG_01271	Isocitrate lyase	753.29 ±130	-	1.296 ± 0.05
		OR643895		442.52 ± 25	-	
	WT	TERG_01281	Malate synthase	2608.72 ± 289	-	7.294 ± 0.75
	Δ*stuA*	TERG_01281	Malate synthase	1009.51 ± 154	-	1.544 ± 0.32
**Keratin**						
**Time**	**Strain**	**Gene**	**Gene Product**	Read Counts *	**Fold Change ¥**	**Enzymatic Activity (U/mg)**
24 h	WT	TERG_01271	Isocitrate lyase	1524.74 ± 79	10 ± 0.003	1.004 ± 0.14
		OR643895		313.06 ± 133	10 ± 0.003	
	Δ*stuA*	TERG_01271	Isocitrate lyase	642.73 ± 77	6.590 ± 0.045	3.528 ± 0.12
		OR643895		196.89 ± 50	4.64 ±0.03	
	WT	TERG_01281	Malate synthase	1284.05 ± 392	1 ± 0.00007	1.397 ± 0.10
	Δ*stuA*	TERG_01281	Malate synthase	683.97 ± 511	0.132 ± 0.020	4.676 ± 0.37
48 h	WT	TERG_01271	Isocitrate lyase	1710.62 ± 207	10 ± 0.005	3.146 ± 0.37
		OR643895		1332.79 ± 897	10 ± 0.002	
	Δ*stuA*	TERG_01271	Isocitrate lyase	546.82 ± 8	2.720 ± 0.042	0.480 ± 0.02
		OR643895		251.66 ± 65	7.98 ± 0.07	
	WT	TERG_01281	Malate synthase	3456.70 ± 2539	5 ± 0.00005	5.691± 0.4
	Δ*stuA*	TERG_01281	Malate synthase	490.55 ± 100	0.380 ± 0.003	5.618 ± 1.0
96 h	WT	TERG_01271	Isocitrate lyase	888.29 ±20	-	1.647 ± 0.05
		OR643895		1436.67 ± 532	-	
	Δ*stuA*	TERG_01271	Isocitrate lyase	365.86 ± 138	-	1.218 ± 0.17
		OR643895		313.63 ± 68	-	
	WT	TERG_01281	Malate synthase	2018.73 ± 815	-	7.824 ± 0.04
	Δ*stuA*	TERG_0128	Malate synthase	622.34 ± 60	-	6.843 ± 0.7

* Number of normalized read counts obtained from a previously published RNA sequencing dataset [23]. ¥ Fold change values obtained in this study by a reverse-transcription quantitative polymerase chain reaction. - Not determined.

## Data Availability

The data presented in this study are available in this article and the accompanying Supplementary Data.

## Supplementary Materials

The following supporting information can be downloaded at https://www.mdpi.com/article/10.3390/ijms25010405/s1.
