# Supplementary material for: The Transcription Factor StuA Regulates the Glyoxylate Cycle in the Dermatophyte Trichophyton rubrum under Carbon Starvation
Source: Int J Mol Sci. 2023 Dec 28;25(1):405. doi: 10.3390/ijms25010405 (PMC10778625; doi:10.3390/ijms25010405)
Supplement: Supplementary file 1 [file ijms-25-00405-s001.zip › ijms-2740558-supplementary.pdf]

International Journal of *Molecular Sciences*

MDPI

Supplementary Material

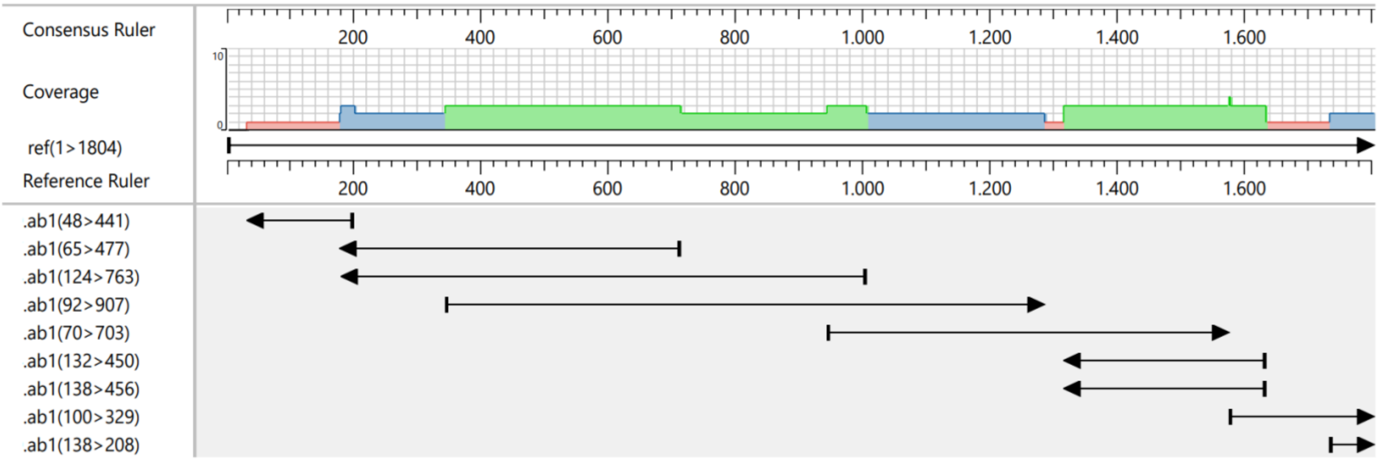

Figure S1. Alignment of cDNA reads (regions flanking the supercontig of TERG_11637, TERG_11638, and TERG_11639) with the reference genome of *Trichophyton rubrum*. The arrows indicate primer orientation, and the bars represent sequencing coverage quality: red for low coverage, blue for intermediate coverage, and green for high coverage. "Ab1" designates the sequencing primers used (listed in table S1).

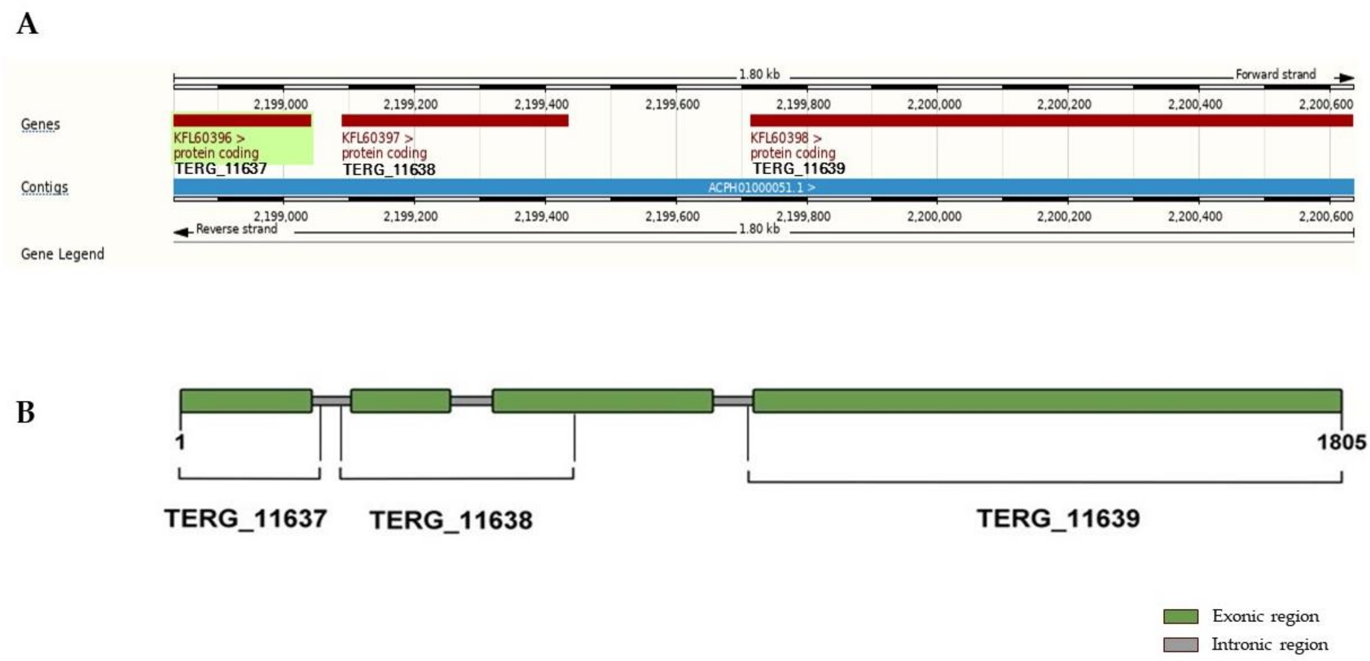

Figure S2. **[A]** Illustration of the supercontig containing TERG_11637, TERG_11638, and TERG_11639 according to the Ensembl Fungi database. **[B]** Schematic representation of the new isocitrate lyase gene (OR643895) obtained from our sequencing results. Brackets indicate the location of each TERG currently annotated in the database.

A

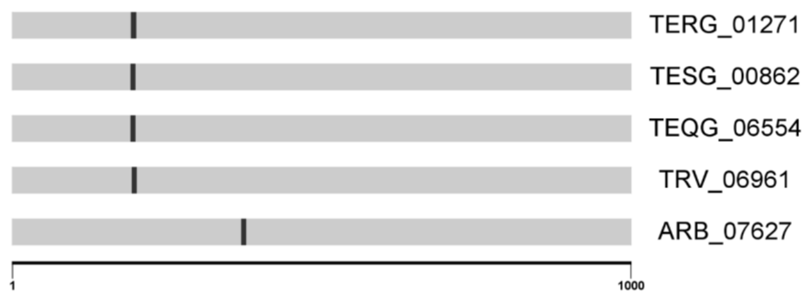

B

```
TERG_01271      CTCAGCGAGCTCGCGAGAAAAGGCGCGGATCTGGGCAGTAGAGGGGACCTGGCCGGTCAA    242
TESG_00862      CTCGGCCAGCTCGCGAGAAAAGGCGCGGACCTGGGCGGTAGAGGGAACCTGGCCGGTCAA    241
TEQG_06554      CTCGGCCAGCTCGCGAGAAAAGGCGCGGACCTGGGCGGTAGAGGGAACCTGGCCGGTCAA    241
TRV_06961       CTCAGCGAGCTCGCGAGAAAAGGCGCGGACCTGGGCGGTAGAGGGGACCTGGCCGGTCAA    243
ARB_07627       CTCAGCGAGCTCGCGAGAAAAGGCGCGGACCTGGGCAGTAGAGGGGACCTGGCCGGTCAA    420
                *** ** ********************** ****** ******** **************
```

Figure S3. [A] Consensus sequence position for StuA in the promoter region of the gene TERG_01271 and its homologs in other dermatophytes. The consensus binding site was determined by identifying DNA sequences upstream (1,000-bp), showing the StuA 5'-[A/T]CGCG[T/A]N[A/C]-3' DNA-binding consensus. **[B]** Alignment of the promoter region from the five dermatophytes, highlighting the StuA consensus sequence. The numbers on the right indicate the position of the last base of each sequence in the alignment. Identification of dermatophytes is denoted as follows: TERG, *Trichophyton rubrum*; TESG, *Trichophyton tonsurans;* TEQG, *Trichophyton equinum*; TRV, *Trichophyton verrucossum*; ARB, *Arthroderma benhamiae*.

Table S1. Primers used in RT-qPCR analysis and DNA sequencing.

Target	Gene Product Name	Primer sequence (5'-3')	Concentration (nM)
TERG_01271	Isocitrate lyase	F: CACACTCTGGGAGCAATCGA<br>R: GGCACGGTATTGTAAGGGTAG	F: 100 nM<br>R: 70 nM
TERG_01281	Malate synthase	F: ACCCCTAACCAACTCTTTGTC<br>R: GAAGCCATCCCTCCATGTAAG	F: 200 nM<br>R: 200 nM
OR643895	Isocitrate lyase	F: CCCTATGAATACTGTGCCGAAC<br>R: TCAGCAATAATCGGACGGAGAT	F: 70 nM<br>R: 100 nM
TERG_01271 (IR-2)	Isocitrate lyase	F: GGGTAAGCAGCATCTTTATC<br>R: GCGCCGTTTTCAGCAAACAACT	F: 200 nM<br>R: 200 nM
gapdh	glyceraldehyde-3-phosphate dehydrogenase	F: GCGTGACCCAGCCAACA<br>R: CGGTGGACTTCGACGATGTAGT	F: 200 nM<br>R: 200 nM
*ab1(92 > 907)	Isocitrate lyase	F: CAGAGCAAAAAGTTGTGGA	2,500 nM
*ab1(124 > 763)	Isocitrate lyase	R: CTGGTTTACACACAACTGCC	2,500 nM
*ab1(48 > 441)	Isocitrate lyase	R: GGATGGCTACAAGTCGGTT	2,500 nM
*ab1(70 > 703)	Isocitrate lyase	F: GCCGAACAAGGTCAATCAGCTT	2,500 nM
*ab1(100 > 329)	Isocitrate lyase	F: CCAACTCTAGCATTGAGCCTCT	2,500 nM
*ab1 (138 > 208)	Isocitrate lyase	F: GAGCAGAAACTGGCATACAACC	2,500 nM
*ab1 (65 > 447)	Isocitrate lyase	R: CCTCTCTAGTTCGTGGCGAAT	2,500 nM
*ab1 (132 > 450)	Isocitrate lyase	R: GGATGGCTACAAGTCGGTTGA	2,500 nM
*ab1 (138 > 456)	Isocitrale lyase	R: AAGCTGATTGACCTTGTTCGGC	2,500 nM

F: Forward Primer- R: Reverse Primer- nM: nanomolar- IR-2: Intron 2 retention- *ab1: Primers used for DNA sequencing.
